# Stakeholder Perceptions on Landscape Governance in Northern Ghana: A Q-Study to Identify Common Concern Entry Points for Integrated Landscape Approaches

**DOI:** 10.1007/s00267-023-01881-2

**Published:** 2023-09-30

**Authors:** Eric Rega Christophe Bayala

**Affiliations:** 1 Amsterdam Institute for Social Science Research (AISSR) / Department of Geography, Planning and International Development Studies, University of Amsterdam, Amsterdam, The Netherlands; 2 Center for International Forestry Research (CIFOR), Bogor, Indonesia

**Keywords:** Perceptions, Common concern entry points, Landscape approach, Q-method, CREMA, Ghana

## Abstract

In a landscape, perceptions can influence people’s actions and behavior toward natural resource use. Improving landscape governance, therefore, requires understanding the different concerns of stakeholders operating within the landscape. This paper analyzes the perceptions of local stakeholders—local landscape users, practitioners engaged in conservation and sustainable resource use, and private actors—regarding the landscape governance system, using the Q-methodology to identify common concern entry points for the implementation of a landscape approach in the Western Wildlife Corridor (WWC) in northern Ghana. To this end, individual interviews and focus groups were conducted with local communities and organizations operating in three Community Resource Management Areas (CREMAs). They identified destructive livelihood activities, constrained livelihoods, and a weak governance system as the main challenges, and the need to balance livelihoods with conservation, strengthening landscape governance through the CREMA initiative, and awareness raising as the main solutions. Thus, the Q-method allowed identifying common concern entry points regarding landscape challenges, governance issues, and potential solutions. I argue that consensus among stakeholders regarding these challenges and solutions could lay the groundwork for a multi-stakeholder process in the area, which could help foster the implementation of an integrated landscape approach in the WWC landscape. It is crucial to support the livelihoods of local people to reduce pressures on natural resources. It is also important to strengthen the functioning of local CREMA management bodies with technical, logistical, and financial support. Implementing a participatory monitoring and evaluation mechanism is critical in this regard.

## Introduction

Facing natural resource degradation, biodiversity loss, climate change, food insecurity, and poverty (Reed et al. 2016; Barlow et al. 2018), the sustainable management of tropical landscapes for conservation and development has become a priority. One of the major challenges of the 21st century is to reduce the duality between biodiversity conservation and livelihoods by considering and negotiating trade-offs between different land uses (Reed et al. 2016, 2020; Omoding et al. 2020b).

The search for a balance between conservation and development objectives has led to an interest in considering various stakeholder perspectives in natural resource governance systems (Buizer et al. 2011; Wali et al. 2017). Indeed, the development of conservation spaces and the governance of multifunctional landscapes require new approaches that integrate the livelihood needs of local people with conservation initiatives (IUCN 2012; Reed et al. 2015). Most rural landscapes in the tropics are multifunctional, providing a habitat for wildlife and local communities, agricultural fields, conservation areas, and economic activities (Freeman et al. 2015). Therefore, the different landscape actors^1^ must have a framework or platform to exchange and negotiate their conservation interests and development priorities (Ravikumar et al. 2018). Integrated landscape approaches (ILAs) are widely recommended for concerted action to negotiate trade-offs between competing land uses (Sayer et al. 2013; Reed et al. 2017). Such approaches aim to balance potentially conflicting land uses by applying “tools and concepts for allocating and managing land to achieve social, economic and environmental objectives” (Sayer et al. 2013, p. 8349). Among the ten principles of landscape approaches, Principle 2 suggests the identification of common concern entry points to encourage dialogue between stakeholders (Sayer et al. 2013). According to Sayer et al. (2013), solutions to landscape problems should be negotiated through trust-based approaches. However, this trust is only built when stakeholders agree on common objectives and values. It is easier to agree on intermediate objectives that are simpler to achieve in the short term, as global objectives are more difficult to reach a consensus on, and this could provide a basis for stakeholders to start working together. Stakeholders only engage in a process when they have an interest in doing so (Bennett 2016), hence the importance of finding their common concerns. This study aims to identify such common concerns in a community-based landscape governance system in Ghana called Community Resource Management Area (CREMA). The Wildlife Division (WD) of the Ghana Forestry Commission adopted and implemented the CREMA in the 2000s to ensure effective and inclusive governance based on a dynamic collaboration between conservation stakeholders and landscape users (Agyare et al. 2015). Despite several challenges in balancing conservation and livelihood needs (Agyare et al. 2015; Ahmed and Gasparatos 2020), the CREMA system offers a promising entry point for implementing a landscape approach (Foli et al. 2018). The principles for a landscape approach suggest good collaboration among all key stakeholders in the landscape, better integration of different and potentially competing land uses, and more inclusivity (Sayer et al. 2013). Collaboration and negotiation are essential characteristics of a landscape approach toward governing forests, landscapes, and the environment as a whole (Opdam et al. 2016). This collaboration is affected by different perceptions of the landscape actors concerned.

Perceptions influence people’s actions and behavior toward the use of natural resources in a landscape (Carmenta et al. 2017). Local populations can consider conservation initiatives as positive or negative, depending on the approach used and the degree of implications. This determines whether they collaborate and take ownership of these initiatives. But also, access or lack of access to the benefits of the initiatives, or the fear of reliving the same failings as those of past initiatives, can influence their decision to participate (Gilli et al. 2020; Omoding et al. 2020a). One could then deduce that collaborative landscape governance can only be a reality when local actors positively perceive the landscape governance mechanism put in place. A negative perception of a natural resource governance system could lead local communities to stop collaborating and ignore initiatives for joint management and sustainable use of resources (Carmenta et al. 2017). Hence the need to understand the different concerns of stakeholders to ensure that they are better taken into account in conservation approaches, as their success or failure depends on it (Bennett 2016; Zabala et al. 2018). Studying local actors’ perceptions helps better understand their judgment of the socio-ecological impacts of conservation and the legitimacy and acceptability they give to the governance of natural resources in their landscape (Bennett 2016). Against this background, this study aims to analyze the perceptions of local stakeholders regarding the CREMA governance system implemented in the Western Wildlife Corridor (WWC) in northern Ghana to identify common concern entry points for implementing a landscape approach.

Several authors have studied the CREMA concept and landscape approaches, but there are hardly any studies that explored them in relation to each other or the potential of the CREMA mechanism for the implementation of a landscape approach (exceptions are Foli et al. 2018; Mansourian et al. 2019; and Hedden-Dunkhorst and Schmitt 2020)—although IUCN recognized this potential as early in 2012 (Nyame et al. 2012). This article aims to contribute to this emerging scholarship by examining the relationship between the CREMA system and landscape approaches from local stakeholders’ perspectives based on field data.

The main question guiding this article is: How do local landscape actors perceive the current landscape regarding conflicting land uses and conflicting conservation-development claims? The first sub-question to answer was: Who are the stakeholders with interest in the WWC landscape? (RQ1). The next two sub-questions focus on stakeholder perspectives: (RQ2) What perspectives emerge from local actors’ views regarding problems and challenges^2^ affecting their landscape and its governance? (RQ3) How do they frame the potential solutions to address these challenges?

The article first presents the methodology adopted for the study. Next, it categorizes the local actors in the study landscape and their interests. This is followed by an analysis of data collected on perceptions and perspectives of the CREMA governance system. Both analyses form the basis for identifying actors’ common concerns and priorities for action. In the final part of the paper, I discuss how addressing the common concern entry points for implementing a landscape approach in the WWC can help improve landscape governance.

## Methodology

### Methodological Approach: The Q Methodology

This study used the Q methodology as it is one of the most appropriate methods to analyze and understand the subjectivity of stakeholders, especially in natural resource governance (Hugé et al. 2016; Sumberg et al. 2017; Zabala et al. 2018; Langston et al. 2019; Lundberg et al. 2020). It is an approach that allows for the understanding and integration of complex aspects of the human dimension, such as actors’ perceptions, beliefs, attitudes, values, and plural perspectives (Zabala and Pascual 2016; Carmenta et al. 2017; Zabala et al. 2018; Tuokuu et al. 2019). The main features of the Q-methodology are threefold: first, differentiating the views of actors by grouping those that are similar; second, presenting the distinct statements to which the participants either strongly agree or strongly disagree; and finally, identifying the consensual statements (Donner 2001; Amaruzaman et al. 2017). Developed in the 1930s by William Stephenson, it combines qualitative and quantitative research techniques (Banasick 2019; Tuokuu et al. 2019; Vaas et al. 2019).

In sum, Q is a tool that is characterized by its capacity to “identify areas of consensus and disagreement around key conservation topics, which can then be used to resolve conflicts, assess management alternatives, appraise policies, or facilitate critical reflection” (Zabala et al. 2018, p. 1193). Hence its use in this study to identify common concern entry points for the implementation of a landscape approach in the WWC in northern Ghana.

**Fig. 1:**
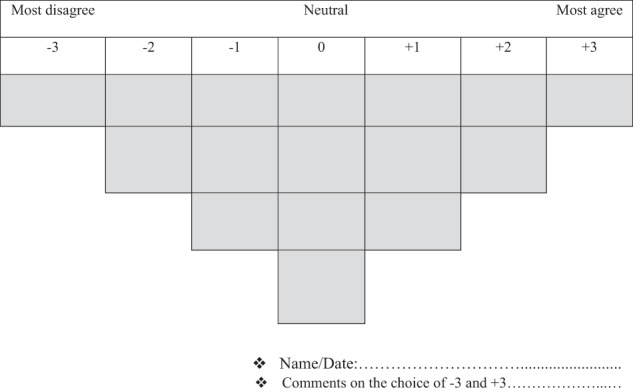
The Q-sort grid displaying the degree of (dis)agreement with the statements, following a quasi-normal distribution. Source: Adapted from Zabala (2014)

A Q-study is a methodological process that begins with collecting statements/affirmations related to the study topic (the Q-set), usually derived from previous studies (Brown 1996; Banasick 2019; Tuokuu et al. 2019). A Q-set is thus a set of possible views related to a specific research question (Sumberg et al. 2017). Each study participant will then rank and sort the statements selected by the researcher according to a bell-shaped grid mimicking a normal distribution predefined for this purpose (Fig. 1). On this grid, the participants have to place the statements they agree with the most on the right (positive signs). In contrast, the statements they disagree with the most are placed on the left (negative signs). The final result of this exercise is called the Q-sort (Sandbrook et al. 2011; Sumberg et al. 2017; Banasick 2019). The next step is to process and analyze the resulting data using appropriate software such as Ken-Q Analysis, PQMethod, and R (Sumberg et al. 2017; Zabala et al. 2018; Langston et al. 2019). For this study, the R software was used.

### The Q-Set Design

Concerning the number of statements to be included in a Q-set, most Q studies have considered a number between 40 and 60, depending on the size of the sorting grid. The number should be such that the participants are forced to rank the statements in a bell-shaped curve to mimic a normal distribution based on which a z-score can distill a single metric (Watts and Stenner 2005; Sumberg et al. 2017). However, a smaller number of statements may be more appropriate when several Q-sets are sorted in one session (as in this study) or in studies involving children (Sumberg et al. 2017). For the present study, two Q-sets were developed based on 12 key informant interviews, 34 focus group discussions, and scientific articles on the study topic (Agyare et al. 2015; Baruah et al. 2016; Baruah 2017; Mansourian et al. 2019; Omoding et al. 2020a). Key informant interviews were held with purposively selected representatives of state agencies, NGOs, district assemblies, CREMA bodies, and traditional chiefs in the study area, using semi-structured interview guides (see Appendix 1 in the supplementary material). The focus group discussions at the community level were organized across the three study CREMAs, involving groups of farmers, pastoralists, forest operators, youth, women, and elders (see Appendix 2 in the supplementary material). Each of these groups consisted of six people. The participants were selected by combining purposive and convenience sampling in consultation with the leader of the Community Resource Management Committee (CRMC). Selection criteria included knowledge of the functioning of the CREMA and availability. Interviews and focus groups are ideal methods for obtaining the full spectrum of relevant views from stakeholders (Tuokuu et al. 2019). They also formed the basis for the stakeholder analysis presented in the results section.

Each Q-set—one on landscape and governance challenges and one on solutions—comprised 16 statements (see Tables 1 and 2). Thus, each respondent had to produce two Q-sorts in one session. Initially, 39 statements were collected for Q-set 1 (related to research question 2) and 28 for Q-set 2 (related to research question 3). These were revised to remove overlaps and enhance clarity.

**Table 1:** Statements on challenges (related to research question 2)

No.	Statements	Categories of statements
1	Cutting down trees for timber, firewood, charcoal production, and farm extension	Problems related to human activities
2	Destruction of crops and vegetation by Fulani pastoralists’ cattle	
3	Siltation of rivers due to the degradation of riverbanks by farming activities	
4	Use of practices that degrade landscape resources, such as bushfires, poisoning of water points for fishing, use of chemicals in farms, and poaching of wild animals	
5	Lack of fertile land and water for farming and pastoral activities	Difficulties related to livelihoods
6	Competition among stakeholders over the use of landscape resources	
7	Insufficient provision of basic social services (schools, hospitals, and drinking water) and veterinary services	
8	No incentives to allow young people to develop non-forestry-related activities	
9	Weak collaboration and communication among landscape stakeholders	Problems related to the landscape governance system
10	Dysfunctioning of the CREMA management committees	
11	Bribing CREMA local leaders to access timber resources	
12	Elite capture of the CREMA initiative by local leaders	
13	Loss of trust in the CREMA system by local communities because of unfulfilled promises	
14	Lack of monitoring and evaluation of the CREMA system	
15	Insufficient knowledge and capacity of local communities to understand and engage in the CREMA system	
16	The CREMA initiative depends very much on projects and NGOs	

Statement 4 brings together several factors of environmental degradation, because they were considered by almost all interviewees as belonging to the same set of exploitation techniques that are harmful to the sustainability of natural resources. Thus, the idea conveyed here is the use of practices that are harmful to the environment by certain populations. We have kept the examples of practices to facilitate the respondents’ understanding of the statement

Source: Key informant interviews and focus group discussions (March–October, 2021); (Agyare et al. 2015; Baruah et al. 2016; Baruah 2017; Mansourian et al. 2019; Omoding et al. 2020a)

**Table 2:** Statements on solutions (related to research question 3)

No	Statements	Categories of statements
1	Reduce the size of protected areas to allow people to have new farms	Livelihoods improvement measures
2	Alternative livelihoods and capacity building in good agro-sylvo-pastoral production practices	
3	Train and support local people to develop income-generating activities in order to reduce their dependence on forests	
4	Facilitate the schooling of children to learn new types of work	
5	Regulate hunting activities	Ecological actions
6	Educate communities on the importance of conservation and restoration	
7	Involve all landscape actors, including the Fulani pastoralists, in natural resource governance	
8	Design a land-use plan to facilitate agricultural, pastoral, and conservation activities	
9	Chase the Fulani pastoralists out of the landscape	
10	Regular renewal of CREMA committee members (CRMC and CEC)	Measures to improve the landscape governance
11	Introduce a good monitoring and evaluation system in the CREMA initiative	
12	Organize the charcoal production and wood collection activity well so that the actors can be monitored	
13	Transparency about the choice of CREMA committee leaders	
14	Raise more awareness in local communities about the CREMA initiative	
15	Create a permanent and inclusive multi-stakeholder platform at the landscape level	
16	Make the CREMA management committees more dynamic, powerful, and organized for greater efficiency	

Source: Key informant interviews and focus group discussions (March-October, 2021); (Agyare et al. 2015; Baruah et al. 2016; Baruah 2017; Mansourian et al. 2019; Omoding et al. 2020a)

The statements in Q-set 1 covered three perception categories: problems related to harmful practices, livelihood impediments, and the CREMA governance system (Table 1). The statements on possible solutions collected and sorted for Q-set 2 also fall into three categories: improving livelihoods, implementing ecological actions, and improving landscape governance (see Table 2).

### Study Site

This research was carried out as part of the COLANDS initiative^3^ (Collaborating to Operationalize Landscape Approach for Nature, Development, and Sustainability) led by the Center for International Forestry Research (CIFOR). This initiative aims to operationalize the landscape approach in tropical landscapes in Indonesia, Zambia, and Ghana (Reed et al. 2020). This study took place in the WWC in northern Ghana, where six villages spread over three CREMAs were involved. These are Fumbisi and Kunyinsa in the Builsa Yenning CREMA; Yizesi and Zukpeni in the Moagduri Wuntanluri Kuwomsaasi CREMA; and Nakong and Kwapun in the Sanyiga Kasena Gavara Kara CREMA. These three out of six CREMAs in the WWC were selected because they already had their certificate of devolution and functional governance structures before the start of this research (2019). Moreover, they belong to different districts, each with its own constitution, rules, and particular challenges, thus allowing a comparison of different contexts. Two communities per CREMA were chosen for a fair representation according to size (one large and one small community^4^), CREMA presence (one community that was the seat of the CREMA Executive Committee (CEC) and one that was not), and distance to the forest reserve (one at the forest fringe and one at larger distance from the forest reserve), with accessibility also playing a role in the selection.

The WWC landscape is located in the savannah ecological zone in Northern Ghana and consists largely of shea parklands. Despite the severe pressure exerted on natural resources by local populations seeking to improve their livelihoods (Braimoh and Vlek 2005; Bayala et al. 2020, 2023), the WWC hosts a rich biodiversity (flora and fauna).

The main ethnicities in the study area include the Dagbamba, Sisala, Dagaba, Kasena, Bulsa, Mamprusi, Wala, Chakali, and Lobi. Smaller ethnic groups include the Hausa, Fulani, and Mossi (MoFA n.d.; Awedoba 2006).

The region presents a conflictual socio-economic context and high vulnerability to climate change, exacerbated by the extreme poverty experienced by most of the rural population (Abdul-Moomin et al. 2016). Living in a tropical climate with a single rainy season and a dry season, these populations strongly depend on natural resources. This puts great pressure on natural ecosystems, leading to the degradation and fragmentation of landscapes (Bouché 2007; Bayala et al. 2023). The main subsistence activity is agriculture (mainly rainfed), complemented by hunting, charcoal production, artisanal mining, livestock rearing, exploitation of forest products, and petty trade (Marchetta 2011; Barlow et al. 2018; Owusu-Ansah 2018; Bayala et al. 2020).

In Ghana, land tenure falls under both state and customary systems (Asare et al. 2013; Osei-Tutu 2017). Traditionally, the conservation of natural resources is regulated by rules based on habits and customs, generally specific to the ethnic group, clan, or tribe (Osei-Tutu 2017; Adeyanju et al. 2021). Conservation practices include sacred groves and taboos (e.g., prohibiting the killing and consumption of particular animals or felling particular plant species (Colding and Folke 1997; Osei-Tutu 2017).

As guarantors of respect for customs and taboos, traditional authorities contribute to biodiversity conservation, for instance, by establishing game and wildlife sanctuaries, such as the Boumfum Sanctuary and the Boaben-Fiema Monkey Sanctuary (WD 2000; Osei-Tutu 2017). The CREMA concept is a natural resource governance model aiming to integrate traditional and modern conservation systems and merge local beliefs and value systems with democratic governance (Asare et al. 2013). However, the WCC faces challenges regarding landscape governance due to the poor functioning of governance bodies, a lack of financial and technical resources, and land-use conflicts between stakeholders (Bayala et al. 2020).

### Participants Administrating the Q-set

In a Q study, the sample size of respondents is not the most important thing, but its diversity is (Zabala 2014; Sumberg et al. 2017). Small samples can give meaningful results (Zabala et al. 2018). Indeed, in some cases, factor groupings produce solid results that are not crucially dependent on the sample size of respondents (Watts and Stenner 2005). According to Zabala et al. (2018), the choice of participants in a Q study is usually made in a non-random way: it is purposeful and involves selection criteria. In this study, the participants included institutional and community actors. Because the Q method is based on ranking statements, having reading and writing skills and a good understanding of English were important selection criteria.

Using purposive sampling, 22 participants were selected to administer the Q-set (Table 3). They were chosen more for their diversity (age, gender, social status, type of activity, and type of organization) than for their representativeness. However, the literacy and command of English criteria did not allow representatives of elders and pastoralists to participate in this stage of the study. Reading and writing in English was a handicap for these actor categories, which should be acknowledged as a limitation of this study (see discussion).

**Table 3:** Participants in the Q-set administration

Type of actors	Number of participants
Public sector	7
Private sector and environmental NGOs	2
Local community (traditional and CREMA leaders, farmers, women, youth)	13
Total	22

At the institutional level, nine government agencies and NGOs were chosen from those who participated in key informant interviews based on their knowledge of and role in the CREMA. Thus, the previously interviewed representatives of these organizations have been included in the sample for this Q study. At the community level, the focus groups enabled the identification of representatives of different groups, taking into account the eligibility criteria mentioned above, gender and age balance, and spread over different resource users.

**Fig. 2:**
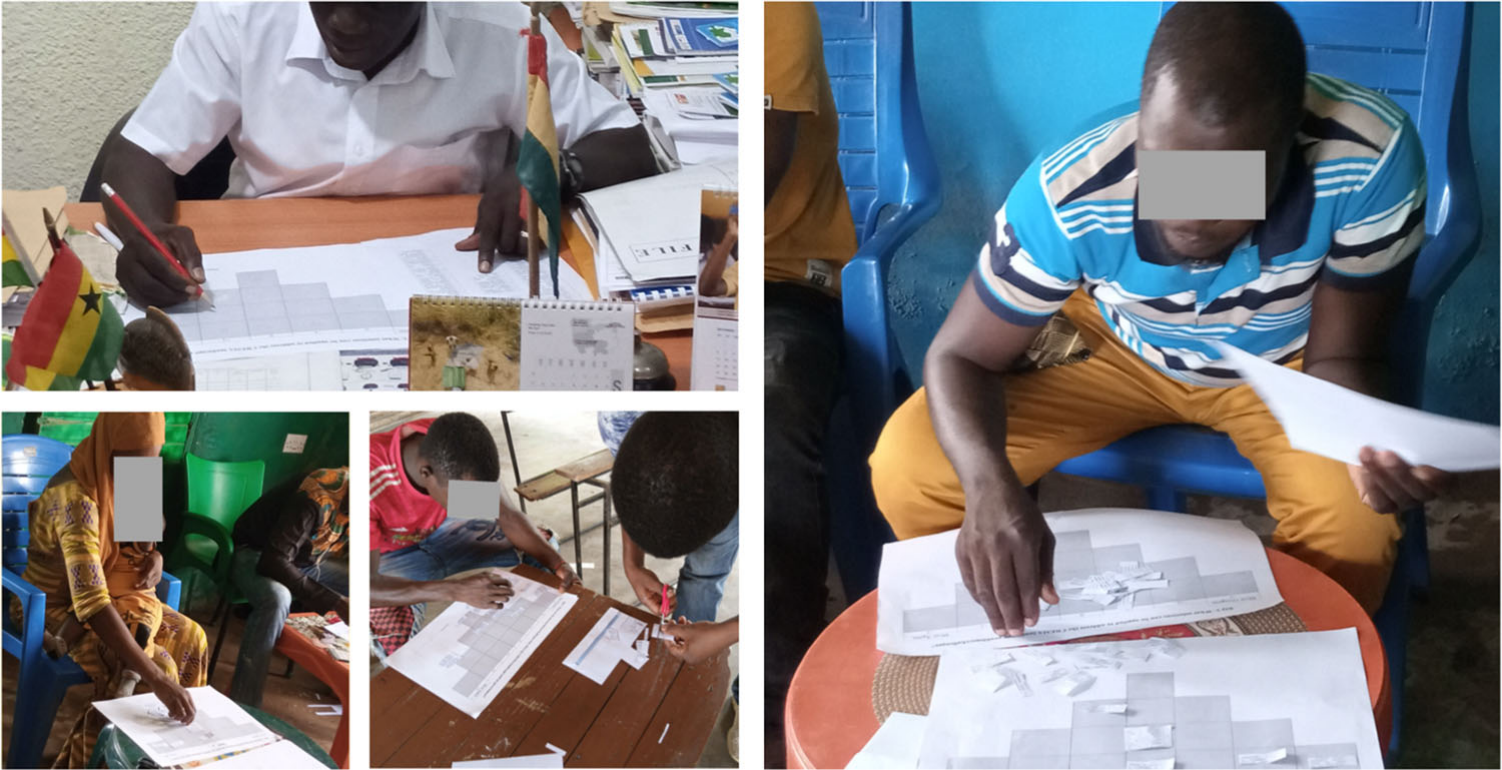
Respondents sorting the statements. Photos: The author, 2021. Source: Field data, 2021

Once the participants were identified, an appointment was made with each of them to administer the Q-set. The purpose of the study was explained, and respondents’ informed consent was granted before they were asked to sort the statements they received into card form. The respondents were instructed to first read all cards for each research question carefully before splitting them into three sets as follows: (i) those with which they agreed; (ii) those with which they disagreed; (iii) and those with which they neither agreed nor disagreed. The statements that were not understood were explained to allow respondents to continue the exercise. The next step was for respondents to place each set of cards on the Q-sorting grid (see Figs. 1 and 2), with the cards with which they most agreed on the right, those they most disagreed with on the left, and the neutral cards in the middle. At the end of this exercise, follow-up interviews were held with each participant, which provided an opportunity to explain the reasoning behind their ranking of the statements (Brown 1980).

### Data Processing

The results of the administration of the two Q-sets were processed and analyzed separately using an Excel spreadsheet and R software (R x 64 4.1.1) and the associated ‘qmethod’ package (version 1.8) (Zabala 2014). First, the data from the Q-sets administration was entered in Excel files, with respondents on the x-axis (rows) and statements on the y-axis (columns). Second, the Excel files were transformed into CSV files before importing them into the R software for the intercorrelations. Third, the principal component analysis module (Sumberg et al. 2017) allowed for calculating intercorrelations and the extraction of factors (common sorting schemes) from the dataset. Fourth, the VARIMAX module was used to rotate the factors and generate factorial solutions. Extracting factors entails condensing all individual responses (Q-sorts) into a few different groupings of responses known as factors, with each factor reflecting one perspective representing respondents with similar views (Zabala and Pascual 2016; Zabala et al. 2018). In Q-analysis, the researcher selects the number of factors to rotate and analyze (Sandbrook et al. 2011). Commonly, these factors are subject to the following criteria (Zabala et al. 2018; Ihemezie et al. 2022): (i) the Kaiser-Guttman criterion, which suggests retaining only factors with an Eigenvalue of 1.00 or higher; (ii) factors with two or more significant factor loadings after extraction (Watts and Stenner 2005); and (iii) the Humphrey rule, which recommends choosing factors whose cross products of the two highest loadings exceed twice the standard error (Brown 1980).

Furthermore, a preliminary interpretation of the factors, considering whether the factor is realistic and the similarities between factors, can also guide the researcher in choosing the number of factors to keep for analysis (Zabala et al. 2018). In similar studies, the number of factors studied generally varies between three and five. The first factors synthesize most of the variability of the initial correlation matrix, which is why these factors are chosen and rotated to obtain a clearer and simpler structure of the data (Sandbrook et al. 2011; Zabala 2014; Buckwell et al. 2020). It is preferable to run different pairs of factors and compare the final results (Webler et al. 2009). Therefore, I made a first rotation based on five factors, then a second based on four factors, and a third considering three factors. Applying the above criteria, three factors were retained for each Q-set (F1, F2, F3 for the Q-set related to RQ2 and F4, F5, F6 for the Q-set related to RQ3) (see Tables 5 and 6). The correlation of the factors for each Q-set revealed the distinctive and consensual statements (see Supplementary material, Appendices 3 and 4) related to each factor. Then, the factor loadings gave insight into the correlation between factors and respondents (Table 7). According to Schober et al. (2018, p1765), “cutoff points are arbitrary and inconsistent and should be used judiciously”. I used a conservative score of 0.7 or higher to flag off components in all responses since the correlations between variables were statistically stronger at this level to explain the association.

## Results

This section first provides a stakeholder analysis and a stakeholder matrix (RQ1). The next two sub-sections present the analyses of interpretations of problems (RQ2) and solutions (RQ3). The last section explores the correlations between factors and stakeholder groups to identify discourse or stakeholder alliances. For ethical reasons, I kept the identities of the study participants anonymous and avoided giving any clues that could help identify them.

### Stakeholder Analysis

To understand landscape dynamics and ensure sustainable natural resource use, stakeholder analysis is key (Reed et al. 2009; Mansourian et al. 2019). Interviews and focus groups were used to analyze the stakeholders, focusing on their typology, interests, and potential in the governance of the three CREMA landscapes studied. Stakeholders are categorized into three groups: local landscape users, government agencies and NGOs, and private actors (see Table 4).

**Table 4:** Stakeholder analysis matrix

Category	Stakeholders	Interest	Asset/potential
Local landscape users	Traditional chiefs	Ensuring the well-being of communities and the sustainability of natural resources	Decision-making power; local leadership
	CREMA leaders (CEC/CRMC)	Conservation and sustainable use of landscape resources	Local landscape governance; community mobilization
	Farmers	Using the land for agriculture	Support for local governance
	Herdmen	Using pasture for livestock	Support for local governance
	Forest resource users	Exploiting forest resources	Support for local governance
Practitioners engaged in conservation and sustainable resource use	Wildlife Division (WD)	Conserving wildlife biodiversity	Administrative authority; promoter of CREMAs
	Forest Services Division (FSD)	Conserving plant biodiversity	Administrative authority; technical support of forest activities
	MoFA (regional office)	Promoting sustainable agriculture	Administrative authority; technical agricultural support
	Environmental Protection Agency (EPA)	Protecting the environment, notably promoting sustainable land and water management	Administrative authority; technical and financial CREMA partner
	Districts assemblies	Local development; Rational use of landscape resources	Decentralized authority; support for the development of CREMAs
	NGO TreeAID	Conserving natural resources and fighting poverty	Technical and financial CREMA partner
	University for Development Studies (UDS) and Center for International Forestry Research (CIFOR)	Scientific research on conservation and livelihoods	Technical and scientific support for the CREMA system
Private actors	Organization for Indigenous Initiatives and Sustainability (ORGIIS)	Sustainable exploitation of non-timber forest products	Technical partner
	Savannah Fruits Company		Technical and financial CREMA partner

Source: Field data, 2021

#### Local landscape users

These are the community actors who depend on natural resources for their survival. In the three CREMAs studied, this group of actors comprised farmers, herders, and forest resource users.

##### Farmers

In northern Ghana, agriculture is the primary source of food and livelihood for local people, most of whom are poor and depend on this activity for survival (Abdul-Moomin et al. 2016; Owusu-Ansah 2018). Farmers thus constitute the majority of stakeholders exploiting the resources of the WWC landscape. Characterized by small-scale, predominantly cereal farms, agricultural activity is predominantly rain-fed and thrives on soil fertility (Owusu-Ansah 2018). However, focus group discussions with farmers revealed that soils have become poor, and the need to conquer new and richer spaces in the landscape has become greater.

##### Herders (pastoralists)

These are also important actors in the study area. They play a major role in the landscape dynamics through clearing, grazing, and uncontrolled bushfires (Saïd and Auvergne 2000). As elsewhere in northern Ghana, livestock activity in the WWC is mainly the domain of Fulani herders who practice pastoralism (Tonah 2006) and have been present in the landscape since the early 20th century (Kuusaana and Bukari 2015; Mensah et al. 2016).

##### Forest Resource Users

Local communities in northern Ghana rely heavily on natural resources—notably charcoal, fuelwood, bushmeat, and non-timber forest products—for their livelihoods (Marchetta 2011). Nationwide, more than 70% of the population depends on forest resources for a part of their income (Amoah and Korle 2020; Baddianaah and Baaweh 2021). In the study area, the exploitation of forest resources is the second most important activity after agriculture and livestock rearing and is equally important for men, women, and youth. The focus groups revealed that using these resources helps make up for constraints on agricultural yields. About 70% of Ghana’s fuelwood and charcoal comes from northern Ghana (Obiri et al. 2014). The exploitation of forests thus constitutes an important income-generating activity for the actors in the area. This specifically applies to women who develop small-scale commercial activities by collecting firewood and producing charcoal (Obiri et al. 2014). They also process shea nuts (*Vitellaria paradoxa*) into butter, the fruits and seeds of the *Parkia biglobosa* into biscuits and cooking ingredients, the fruits of the baobab (*Adansonia digitata*) into biscuits, and tamarind (*Tamarindus indica*) into sirup (Bayala et al. 2020).

In short, the findings reveal that the main interest of local landscape users is to provide for their families and secure their livelihoods, although they also want to conserve and sustainably use their natural resources. The landscapes of northern Ghana offer various natural resources, thus providing users’ primary source of livelihood and socio-economic development.

#### Practitioners engaged in conservation and sustainable resource use

This category consists mainly of staff of public agencies and environmental NGOs. These include the Forestry Commission represented by the WD and the Forest Services Division (FSD), the Environmental Protection Agency (EPA), the regional office of the Ministry of Food and Agriculture (MoFA), the district assemblies, and the NGO TreeAid. This category also includes researchers from the University for Development Studies (UDS) and CIFOR. Their main objective is to conserve the natural ecosystems in the landscape while promoting the sustainable use of these resources by local people to sustain their livelihoods. Hence their interest in promoting community-based landscape management initiatives such as the CREMAs, sustainable agriculture, beekeeping, and poverty alleviation. *“In any conservation initiative, there must be a component for livelihood improvement”*, said interviewee 6.^5^ Ultimately, sustainable natural resource management is the goal.

#### Private actors

This group comprises the NGO ORGIIS (Organization for Indigenous Initiatives and Sustainability) and the Savannah Fruits Company. Their primary aim is to exploit non-timber forest products commercially. They work with local women’s and youth cooperatives, from whom they buy the products for further processing or trade. At the same time, they promote income-generating activities for local communities and support initiatives for the conservation and sustainable use of natural resources, as this guarantees the sustainability of their activities and business. This is why they often develop capacity-building programs for local people related to the sustainable use of natural resources.

Table 4 provides a summary of all the stakeholders identified during the study. It presents the various interests of these stakeholders and their role in the study area. The stakeholder analysis shows that even though the relationships between them are often characterized by conflicting interests (landscape as a source of livelihood, business, or biodiversity conservation), the three stakeholder categories share a common interest in ensuring the sustainability of landscape resources. This commonality could benefit concerted efforts to address the challenges in multifunctional/tropical landscapes. Such multi-stakeholder efforts are needed as landscape governance transcends scales, both horizontally (between various stakeholders) and vertically (from the local to national level) (Mansourian et al. 2019).

The following section presents the stakeholder perceptions of landscape and governance challenges and possible solutions to these problems based on an analysis of the different factors resulting from Q-sorts.

### Interpretation of Factors Related to Landscape and Governance Challenges

Table 5 presents the three factors derived from the statements related to research question 2 (RQ2) about “the main problems/challenges that affect the CREMA landscape and its governance”. The three factors presented in Table 5 lead to the following interpretations:

**Table 5:** Statements and factors related to landscape and governance challenges (RQ2)

No	Statements	F1	F2	F3
1	Cutting down trees for timber, firewood, charcoal production, and farm extension	3	3	−1
2	Destruction of crops and vegetation by Fulani pastoralists’ cattle	2	2	2
3	Siltation of rivers due to the degradation of riverbanks by farming activities	1	1	0
4	Use of practices that degrade the landscape resources, such as bushfires, poisoning of water points for fishing, use of chemicals in farms, and poaching of wild animals	2	2	1
5	Lack of fertile land and water for farming and pastoral activities	−3	1	−1
6	Competition among stakeholders over the use of landscape resources	−2	0	0
7	Insufficient provision of basic social services (schools, hospitals, and drinking water) and veterinary services	−2	0	3
8	No incentives to allow young people to develop non-forestry-related activities	0	0	0
9	Weak collaboration and communication among landscape stakeholders	0	0	−3
10	Dysfunctioning of the CREMA management committees	−1	−1	2
11	Bribing CREMA local leaders to access timber resources	1	−2	−2
12	Elite capture of the CREMA initiative by local leaders	−1	−1	0
13	Loss of trust in the CREMA system by local communities because of unfulfilled promises	−1	−2	−1
14	Lack of monitoring and evaluation of the CREMA system	0	−1	−2
15	Insufficient knowledge and capacity of local communities to understand and engage in the CREMA system	1	−3	1
16	The CREMA initiative depends very much on projects and NGOs	0	1	1

Source: Field data, 2021

#### Factor 1 (F1): Destructive livelihood activities

This factor shows that respondents perceive the main problems and challenges affecting the landscape and its governance to be related to livelihood activities leading to the degradation of natural resources: tree cutting for timber, firewood, charcoal production, and farm extension (score +3); destruction of crops and vegetation by Fulani pastoralists’ cattle (+2); and siltation of rivers due to the degradation of riverbanks by farming activities (+1). Bribing local CREMAs leaders to access timber resources and insufficient knowledge and capacity of local communities to run the CREMA system appear as elements that contribute to undermining the governance and sustainability of the landscape, but most scores regarding general or CREMA governance failures indicate neutrality or disagreement (see Table 5). The score of +3 for statement 1 reflects that all respondents agree that this is the most important problem in the landscape, hence the label “Destructive livelihood activities” for this factor.

This factor does not display concern about the lack of fertile land and water for farming and pastoral activities. The score of −3 (most disagree) indicates that this issue is not seen as a major landscape and governance challenge. In this regard, respondent 11 stated, “*The problem is not the lack of fertile land, but the misuse of available land. If good agricultural practices are applied on the plots being farmed, people will no longer talk about the lack of fertile land*”.^6^

In summary, regarding the major constraints affecting the landscape and its governance, factor 1 highlights destructive natural resource use practices in correlation with bribing local CREMA leaders and insufficient knowledge and capacity to run the CREMA system. Thus, the perceived problems are mostly linked to activities carried out by local people to improve their well-being and, to some extent, to flaws in the governance system.

#### Factor 2 (F2): Constrained livelihoods

This factor shows a similar tendency to factor 1 regarding agreeing with statements on destructive livelihood activities (statements 1–4) and disagreeing with statements on failing CREMA management and leadership (statements 10–13). However, unlike factor 1, respondents agree with the statements about the lack of fertile land and water for farming and pastoral activities and the strong dependence of CREMAs on projects and NGOs. Disagreement with the statements on bribing, the lack of monitoring and evaluation, and insufficient knowledge and capacity of local communities to understand and engage with the CREMA system indicate no concern with the CREMA governance system. Neutrality regarding the statements on the insufficient provision of basic social and veterinary services, the lack of incentives for young people to develop non-forest-related activities, and the weak collaboration and communication among landscape stakeholders indicate little concern with the lack of a supportive governance system.

In sum, factor 2 refers mainly to destructive and constrained livelihood activities as landscape challenges. Regarding governance challenges, this factor reflects a concern only with the donor dependence of the CREMA system.

#### Factor 3 (F3): A weak governance system

Factor 3 corresponds with agreement on the statement regarding the insufficient provision of basic social services by the public administration (+3), the impact of pastoral activities on the landscape (+2), and the malfunctioning of local CREMA governance bodies (+2). These constraints are correlated with the weak capacity and lack of knowledge of local populations, the strong dependence of the CREMA initiative on external partners, and the harmful practices adversely affecting natural resources. Hence, the tendency shown by this factor reveals that the difficulties related to the landscape governance system are seen as the most pertinent ones. On this subject, interviewee 1 expressed himself in these terms: *“The local communities are not yet sufficiently capable of managing the CREMA system. They have not yet fully understood the system and lack the means to make it work. Asking one part of this population to guide the others is like asking blind people to guide other blind people*”.^7^ For respondent 14, *“the local population lacks the capacity to understand and manage the CREMA concept”*.^8^ This reflects the need for capacity building in local communities on the CREMA concept. Understanding the concept is the first step towards its successful implementation (Bempah et al. 2019).

Despite the concerns about weak CREMA governance, this factor displays strong disagreement with statements regarding weak collaboration and communication among landscape stakeholders (−3). Also, disagreement with statements on the lack of monitoring and evaluation of the CREMA system, bribing CREMA local leaders to access timber resources, and loss of trust in the CREMA system by local communities because of unfulfilled promises is somewhat surprising considering the concerns with weak CREMA governance.

### Interpretation of the Factors Related to Solutions

The statements related to research question 3 (RQ3) on “solutions that can be applied to address the CREMA landscape problems/challenges” generated the factors presented in Table 6. The following interpretations are derived from the three factors presented in Table 6.

**Table 6:** Statements and factors related to solutions (RQ3)

No	Statements	F4	F5	F6
1	Reduce the size of protected areas to allow people to have new farms	−3	−2	0
2	Alternative livelihood and capacity building in good agro-sylvo-pastoral production practices	3	−1	0
3	Train and support local people to develop income-generating activities in order to reduce their dependence on forests	2	3	3
4	Facilitate the schooling of children to learn new types of work	0	0	−2
5	Regulate hunting activities	−2	−2	0
6	Educate communities on the importance of conservation and restoration	1	2	2
7	Involve all landscape actors, including the Fulani pastoralists, in natural resource governance	1	1	−1
8	Design a land-use plan to facilitate agricultural, pastoral, and conservation activities	2	0	−1
9	Chase the Fulani pastoralist out of the landscape	−2	−3	0
10	Regular renewal of CREMA committees members (CRMC and CEC)	−1	1	1
11	Introduce a good monitoring and evaluation system in the CREMA initiative	0	1	1
12	Organize the charcoal production and wood collection activity well so that the actors can be monitored	−1	−1	−2
13	Transparency on the choice of CREMA committee leaders	−1	0	−1
14	More awareness in local communities about the CREMA initiative	1	0	2
15	Create a permanent and inclusive multi-stakeholder platform at the landscape level	0	−1	−3
16	Make the CREMA management committees more dynamic, powerful, and organized for greater efficiency	0	2	1

Source: Field data, 2021

#### Factor 4 (F4): Balance livelihoods with conservation

This factor reveals strong agreement with supporting people through alternative livelihood projects and capacity building in good agro-sylvo-pastoral production practices (+3) and income-generating activities (+2). At the same time, this factor reflects the importance attached to conservation: the development of income-generating activities would reduce pressure on forests, while land-use planning could balance conservation with agriculture and pastoralism (+2). Proponents of this factor suggest that communities should be educated on the importance of conservation and restoration (+1), all landscape actors, including the Fulani pastoralists, should be involved in natural resource governance (+1), and local communities should be made more aware of the CREMA initiative (+1).

In sum, this factor suggests that the challenges in the WWC landscape can best be addressed by simultaneously considering conservation and livelihood development.

#### Factor 5 (F5): Strengthen landscape governance through the CREMA initiative

Proponents of this factor foreground the solution related to strengthening the capacity of local communities to develop income-generating activities that make them less dependent on forests (+3) but score negatively on other livelihood-related statements. Instead, they emphasize the importance of improving landscape governance by building the capacity^9^ of communities on conservation and restoration, improving the functioning of CREMA management committees, involving all landscape actors, including the Fulani pastoralists, in natural resource governance, constant renewal of CREMA committee members, and introducing a sound monitoring and evaluation system in the CREMA initiative. Hence, an important role is attached to the CREMA initiative as a means to improve landscape governance; this factor is neutral about designing a land-use plan or regulating charcoal production and wood collection and negative about regulating hunting. This factor displays no concern with the choice and functioning of CREMA leaders but emphasizes the importance of making the CREMA management committees more dynamic, powerful, and organized for greater efficiency (+2).

This factor suggests that improving CREMA governance will positively affect livelihoods. This aligns with the objective of the CREMA concept to ensure better landscape governance for biodiversity conservation while improving the livelihoods of local communities (Agyare 2013; Agyare et al. 2015).

#### Factor 6 (F6): Raise knowledge awareness but forget about stakeholder mobilization

This factor shows many similarities with factor 5 regarding the proposed solutions. The statements that obtained positive scores converge towards prioritizing developing income-generating activities (+3) and strengthening the CREMA governance system through the regular renewal of the CREMA management bodies, strengthening their effectiveness, and implementing a monitoring and evaluation system (+1) (Table 6).

However, two differences come to the fore. The first is a stronger disagreement with proposals related to stakeholder mobilization. This factor scores more negatively than factor 5 on proposals to organize charcoal producers and wood collectors (−2) and stakeholder platforms (−3) and also scores negatively (−1) on the need to engage landscape actors in natural resource governance. Second, like factor 5, this factor reflects a concern about raising awareness of the importance of conservation and restoration among local communities. However, in contrast with factor 5, this must go hand in hand with creating greater awareness of the CREMA initiative.

Thus, the three factors F1 (Destructive livelihood activities), F2 (Constrained livelihoods), and F3 (A weak governance system) reflect the categorization of the statements made in Table 1, with F1 corresponding to the category of statements on “Problems related to human activities”; F2 corresponding to the category on “Difficulties related to livelihoods”; and F3 to “Problems related to the landscape governance system”. Similarly, the factors F4 (Balance livelihoods with conservation), F5 (Strengthen landscape governance through the CREMA initiative), and F6 (Raise knowledge awareness but forget about stakeholder mobilization) in Table 2 coincide respectively with the categories “Livelihoods improvement measures”; “Measures to improve the landscape governance”; and “Implementing ecological actions”.

### Correlations Between Factors and Respondents

Based on correlations between study participants and factors, factor loadings reveal how respondents’ views score on each factor (Table 7). Correlation coefficients are scaled from −1 to +1, where 0 indicates a negligible correlation (Schober et al. 2018). The higher the factor loading score, i.e., tending toward −1 or 1, the stronger the correlation of participants with the factors they represent, with negative scores indicating disagreement and positive scores indicating agreement. The results of these loadings allow identifying conflicting and shared visions and discourse alliances (Di Masso and Zografos 2015; O’Riordan et al. 2019).

**Table 7:** Q-sort factor loadings for problems and challenges (RQ2 and RQ3)

Respondent	Problems (RQ2)			Solutions (RQ3)		
	F1	F2	F3	F4	F5	F6
R1 (Local landscape users/farmer 1)	0.218	0.41	**0.70**	−0.235	0.45	0.68
R2 (Local landscape users/woman 1)	−0.032	−**0.90**	−0.10	0.003	0.61	0.56
R3 (Local landscape users/youngster 1)	0.272	−0.12	−0.50	−0.252	−0.29	0.55
R4 (Local landscape users/youngster 2)	**0.712**	0.11	−0.05	0.241	**0.79**	−0.08
R5 (Local landscape users/woman 2)	−**0.844**	0.17	−0.09	−0.054	0.14	**0.71**
R6 (Local landscape users/youngster 3)	0.696	0.16	0.20	0.684	0.34	−0.11
R7 (Local landscape users/woman 3)	**0.729**	−0.01	−0.20	0.364	0.69	0.26
R8 (Local landscape users/farmer 2)	**0.715**	0.11	0.30	0.184	**0.84**	0.15
R9 (Local landscape users/farmer 3)	0.073	−0.20	**0.80**	0.073	0.54	0.10
R13 (Local landscape users/CEC1)	0.453	0.32	−0.20	0.052	0.13	0.56
R18 (Local landscape users/CEC2)	0.681	0.27	0.30	−0.596	0.15	0.25
R19 (Local landscape users/CEC3)	0.074	**0.73**	0.20	0.216	**0.76**	0.23
R21 (Local landscape users /chief)	0.242	0.47	−0.30	0.476	0.07	0.65
R10 (Private actor)	0.358	−0.10	0.50	0.431	0.30	0.07
R11 (Practitioners engaged in conservation and sustainable resource use/Agric. officer)	0.139	**0.88**	0.10	**0.874**	0.33	−0.03
R12 (Practitioners engaged in conservation and sustainable resource use/EPA)	**0.808**	0.29	−0.02	**0.836**	0.16	0.27
R14 (Practitioners engaged in conservation and sustainable resource use/WD)	0.599	0.44	0.10	0.096	0.25	0.62
R15 (Practitioners engaged in conservation and sustainable resource use/FSD 1)	0.140	0.62	0.40	**0.784**	0.35	−0.18
R16 (Practitioners engaged in conservation and sustainable resource use/FSD 2)	0.486	0.69	−0.0003	0.662	0.14	0.14
R17 (Practitioners engaged in conservation and sustainable resource use/District Ass. District A)	−0.141	0.47	−0.40	0.556	−0.13	**0.71**
R20 (Practitioners engaged in conservation and sustainable resource use/District B)	0.069	0.10	**0.70**	0.548	0.24	0.48
R22 (Practitioners engaged in conservation and sustainable resource use/NGO)	−0.006	−0.20	−**0.80**	0.174	0.01	0.03

*CEC* CREMA Executive Committee, *District Ass* District Assembly, *EPA* Environmental Protection Agency, *FSD* Forest Services Division of the Forestry Cmmission, *NGO* non-governmental organization, *WD* Wildlife Division of the Forestry Commission. Figures in bold indicate a strong (0.70–0.89) or very strong (0.90–1.00) correlation (Schober et al. 2018)

Source: Field data, 2021

The factor loadings on landscape problems/challenges reveal that the discourse reflected in factor 1 that the main problems of the landscape are related to destructive livelihood activities is strongly shaped by both local landscape users and practitioners engaged in conservation and sustainable resource use. But the dominant scores show conflicting views across different actors. Some scores show disagreement, with a strong score of disagreement from woman 2 (−0,84), while three others strongly agreed (youngster 2 (0,71), woman 3 (0,72), farmer 2 (0,71)), same with one actor engaged in conservation and sustainable resource use (EPA (0,8)).

Factor 2 (constrained livelihoods) is also dominated by the same types of actors, with some divergence of views. Indeed, some local landscape users have a very strong negative correlation with the factor (woman 1 (−0,9)), thus expressing a very strong disagreement with the supported discourse, while others of the same group express a strong agreement (CEC3 (0,73)), sharing the same opinion as some practitioners engaged in conservation and sustainable resource use (agricultural officer (0,88)). Similarly, in factor 3 (a weak governance system), the correlation is strongly linked to both actor groups. Three dominant scores indicate agreement from farmer 1 (0,70), farmer 3 (0,8), and district B (0,70), while one actor engaged in conservation and sustainable resource use strongly disagrees (NGO (−0,8)).

The three factors (F1, F2, F3) reflect the discourses of local landscape users and practitioners engaged in conservation and sustainable resource use. While there may be some disagreements, the trends that emerge from the factors find support from most respondents in these two stakeholder groups.

Regarding the factors relating to solutions, factor 4, which reflects the discourse that the sustainability of the WWC landscape must be based on a balance between livelihoods and conservation, is strongly correlated with the views of practitioners engaged in conservation and sustainable resource use (agricultural officer (0,87), EPA (0,83) and FSD 1 (0,78)); while factor 5 (strengthen landscape governance through the CREMA initiative) is strongly correlated with those of local landscape users (youngster 2 (0,79), farmer 2 (0,84) and CEC 3 (0,76)). Factor loadings for factor 6 (raise knowledge and awareness but forget about stakeholder mobilization) again show influence from both practitioners engaged in conservation and sustainable resource use (District assembly A (0,71)) and local landscape users (women 2 (0,71)), indicating agreement on the actions required to solve landscape challenges.

However, the correlations between the different factors allowed the identification of consensus statements between the different Q respondents.

### Common Concern Entry Points for the Implementation of a Landscape Approach

Consensus between stakeholders on the main problems and possible solutions could be entry points for implementing a landscape approach in the WWC. Indeed, one of the ten principles of the landscape approach (principle 2) suggests that landscape stakeholders should have one or more common concerns (Sayer et al. 2013) that can facilitate their coming together in a common framework for reflection, dialogue, and decision-making, in order to find a common solution(s). Thus, this study considers four consensus points as common concern entry points for implementing an ILA in the WWC. They are further elaborated on below.

#### Common concern entry point 1: siltation and drying up of water bodies

The stakeholders are unanimous on the fact that the agricultural activities carried out on the riverbanks contribute to the siltation of these water points, hence a rapid drying up of the resource. Focus groups with farmers, pastoralists, forest operators, women, youth, and elders revealed that the water problem is crucial in the study area. The early drying up of water points makes the populations highly dependent on rainfall for agricultural and pastoral production. Water is vital for people and their livelihood activities, but access to potable water and water for irrigation has become a problem for most rural communities (Bazaanah and Dakurah 2021). *“The water bodies dry fast now. After the rainy season, we have problems getting water*”, the young people of the CREMA of Builsa Yenning expressed during the focus group discussions.^10^

The permanent availability of water would allow the development of off-season activities in the study area, thus reducing the dependence of local communities on forests and conservation areas for their livelihoods. This is why water reservoirs are in high demand by rural people in the WWC and northern Ghana in general, because of the multiple benefits for irrigation, livestock, fishing, and brick-making (Acheampong et al. 2018). Thus, the degradation of water sources hinders livelihoods and threatens the conservation of natural resources in the WWC landscape, especially in a context marked by the adverse effects of climate change. A study conducted by Glitse et al. (2018) highlights the degradation of the livelihoods of local communities due to climate change which is causing open water reservoirs in northern Ghana, specifically in the Upper East Region, to dry up. Therefore, the same study suggests exploring new water storage technologies, such as the Bhungroo^11^ technology, which stores rainwater underground. This would have the advantage of minimizing evaporation and making water available all year round to facilitate dry season cropping (irrigation), although this area also has many other challenges to overcome, such as land tenure problems, encroachment, lack of technological know-how, high input costs, etc. (Glitse et al. 2018). However, the ongoing political initiative of the Government of Ghana entitled “Infrastructure for Poverty Eradication Program”, of which one of the projects is “one village, one dam”, could be a helpful solution to the water problem in northern Ghana if it is successful (Ghansah et al. 2022).

#### Common concern entry point 2: financial constraints to the CREMA initiative

Consensus exists on the limited viability of the CREMAs initiative to facilitate landscape governance in the WWC due to financial constraints. This observation was widely discussed during the interviews and focus groups. The strong dependence of local landscape management bodies (CRMC and CEC) on externally financed projects and NGOs is a point of concern, as it limits the effectiveness of the governance system put in place when project funding comes to an end. Interviewee 3 believes that *“the CREMA system is good, but not functioning due to financial constraints”*,^12^; and according to interviewee 4: *“the CREMA system is good, but it needs to be improved”*.^13^ These are testimonies to the hope placed in the CREMA system to improve the living conditions of the people and strengthen the conservation of natural resources, and financial autonomy could allow the mechanism to function better and achieve the desired objective.

In many cases, the CREMA system and its governance bodies were established through short-term projects whose support faded even before the system was fully functional and the governing committees were technically and financially capable of taking over (Agyare 2017). This is the case of the CREMAs covered by this study, whose establishment was prompted by the EPA through the Sustainable Land and Water Management Project, which lasted only five years (2016–2020). The non-implementation of an exit plan to ensure the sustainability of the CREMA management bodies has led to their dysfunction. Left on their own without financial support, they struggle to carry out the tasks they were created for. Agyare (2017) also suggests that measures to revitalize these local landscape management bodies are needed to ensure functional leadership and good governance within the CREMAs. Among these measures, implementing medium or long-term action plans supported by the CREMA initiators and their partners could help ensure the long-term functionality of the CREMA system.

#### Common concern entry point 3: the need for livelihood support

The advantages in terms of livelihood that local communities benefit, or can benefit from, are the main factors that condition their engagement in the landscape governance system, including the CREMA system (Abukari and Mwalyosi 2018; Baddianaah and Baaweh 2021). The study revealed that local communities expect their living conditions to improve through the CREMA system. They will ensure it works well as long as they get tangible benefits from the system. This aligns with a study on the Zukpiri CREMA in the Upper West Region of Ghana (Baddianaah and Baaweh 2021). This is reflected in stakeholders’ agreement on the need to promote livelihood diversification and create alternative income-generating activities through capacity building, reducing the pressure on natural resources. Interviewee 6 considers that *“The CREMA system is one of the best ways to protect the landscape. But it is important to tie the livelihoods of people to conservation objectives. Also, the lack of monitoring in the system is a big issue”*^14^. It is, therefore, not surprising that there is consensus on statement 11.

#### Common concern entry point 4: the need for monitoring and evaluation

According to the respondents, monitoring and evaluation are a component of landscape governance that is missing in the study area. *“A good monitoring system is needed in the CREMA process; the Forestry Commission should monitor the system”*, according to interviewee 1.^15^ This is why, by consensus, the respondents consider the implementation of a solid and adequate monitoring and evaluation mechanism for the CREMA initiative as an ultimate solution for the proper functioning of the landscape governance system. Monitoring and evaluation are considered essential to assess the functioning of a project, initiative, or system to improve its results (Kariuki 2014; Kabonga 2019). Its implementation as a continuous process could be beneficial in reframing and orienting management and governance activities, dynamizing management and governance bodies, and promoting accountability and transparency in the functioning of CREMAs. In short, it helps to identify the deficiencies of the mechanism in terms of people’s livelihoods and conservation to find solutions to remedy them. This meets the interests of both local landscape users and practitioners engaged in conservation actions. To strengthen the commitment of all stakeholders, adopting a participatory monitoring and evaluation model could elicit the active engagement of key stakeholders. Additionally, Chervier et al. (2020)^16^ suggest that landscape governance practitioners consider a mixed method that combines monitoring tools and evaluation of the effectiveness of the system in place through process analysis or impact assessment, especially in the context of a landscape approach.

## Discussion

### Assets and Constraints for Landscape Approach Implementation

The results of this study show that in the WWC landscape, three different stakeholder groups, namely local landscape users, practitioners engaged in livelihoods and sustainable resource use, and private actors, co-exist, each operating according to its interests. Despite being subject to the same landscape dynamics, they perceive the problems and how to solve them differently. Perceptions are shaped by personal experiences and individual interests. This leads people to prioritize challenges and their solutions differently. What is perceived as a priority problem or solution by some is seen as less urgent by others.

The study of these different viewpoints has allowed the identification of consensual understandings among stakeholders of concerns and solutions that could form a basis for implementing a landscape approach. It will be easier to engage stakeholders around issues they already agree on; stakeholders only join a process when they judge it to be in their interest (Sayer et al. 2013; Bennett 2016; Carmenta et al. 2017). Consensus constitutes the basis for collaborative work and multi-stakeholder processes needed for implementing a landscape approach (Sayer et al. 2013). In Uganda, the implementation of an ILA in the Agoro-Agu landscape required seeking a consensus on “balancing the competing interests in the landscape”, which constituted the common concern entry point (Omoding et al. 2020b, p102). In this paper, I argue that the consensus perceptions identified through the Q-method can be seen as common concern entry points for bringing stakeholders with diverging interests into a multi-stakeholder platform and initiating a multi-stakeholder process at the WWC level.

The four consensus elements focus on livelihood improvement and strengthening the landscape governance system. Despite different perceptions and objectives, a focus on common concern entry points implies that each stakeholder will be interested in collaborating on these issues. In the present context, the interest of local communities in joining a multi-stakeholder process is related to improving their capacity to provide for their food needs and taking responsibility for ensuring the sustainability of landscape resources. For conservation actors, the effective functioning of CREMAs implies more effective biodiversity conservation. This is also in the interest of private actors engaged in the trade of non-timber forest products.

Furthermore, apart from the elements of consensus, respondents often have convergent views on several other statements. For instance, most respondents entirely refuted the ‘solution’ that the Fulani pastoralists should be chased out of the landscape (through a score of −2 (F4) and −3 (F5)). This is certainly due to the complexity of the Fulani pastoralists issue in Ghana (see Tonah 2006; Bukari and Schareika 2015; Kuusaana and Bukari 2015; Bukari et al. 2018). In addition, statements regarding the reduction of protected areas, the control of hunting, alternative livelihoods and agro-sylvo-pastoral capacity building, the organization of charcoal production and wood collection, and the creation of a permanent and inclusive multi-stakeholder platform at the landscape level are solutions with which many respondents expressed disagreement. This means that these solutions are not priorities. The fact that most actors interviewed are often unanimous on certain issues implies that they have more convergent than divergent opinions on the solutions to the problems of their landscape. This is an asset in the search for trade-offs. However, their disagreement on solutions to mobilize stakeholders, to better organize the use of resources, and create a multi-stakeholder platform could constitute a handicap for a multi-stakeholder process and consequently implement a landscape approach.

Perceptions constitute a crucial source of information that can help strengthen landscape governance mechanisms (Bennett 2016; Omoding et al. 2020a). Indeed, data from perceptions can help to refine strategies for implementing conservation and development initiatives, especially through better alignment of awareness-raising and capacity-building interventions and informed design of governance tools (Kotowicz et al. 2017). Case studies by Walters et al. (2021) on restoration initiatives in Africa confirm this through examples of projects that have failed or have been redesigned due to a lack of knowledge or consideration of local perceptions. Furthermore, in a study of a protected area, Webb et al. (2004) found that using stakeholder perceptions is an affordable but powerful way of assessing the performance and impacts of protected area management.

This study of perceptions has provided insight into the views of stakeholders on landscape problems and potential solutions, but more importantly, it has provided an understanding of the different discourses that shape the WWC landscape, as well as the consensual views of stakeholders. All these data are valuable to feed eventual policies and strategies for implementing integrated landscape governance approaches and fostering evidence-based conservation (see Sutherland et al. 2004; Bennett 2016). Indeed, in the context of ILAs, understanding stakeholder perceptions and applying them appropriately is an efficient way of improving landscape governance (Omoding et al. 2020a). Therefore, I argue that perceptions should be considered strategic data sources that contribute to better planning and ensure the sustainability of landscape resources through strong local involvement. In the same vein, Walters et al. (2021) advocate greater consideration of local values and knowledge, including stakeholder perspectives, in landscape governance initiatives. After all, the success of conservation efforts usually depends on the support of local communities, which is greatly influenced by perceptions of the effects on the community and attitudes about governance (Bennett and Dearden 2013).

### Proposals for Improving Livelihoods and Strengthening Landscape Governance

Several recommendations emerge from this study. First, as initiators of the CREMA, the Forestry Commission and EPA should seek the support of NGOs and private sector actors in the area to identify opportunities for improving livelihoods. Such opportunities include training in small-scale activities such as welding, carpentry, and processing and trading non-timber forest products such as shea butter, *dawadawa*^17^, and honey. Ecotourism and cultural tourism could also be explored as potential income-generating activities—both directly (work as guides and eco-guards) and indirectly (production and sale of carved objects and pottery). The resulting revenue could contribute to the functioning of the CREMA management bodies.

Second, the CREMAs initiators, together with the district assemblies and other development partners, should encourage and stimulate water control projects (small reservoirs) and potable water supply. Technologies adapted to the climatic context of Northern Ghana would be more appropriate. This may also create opportunities for food production in home gardens and fodder production that could help reduce human pressure on the natural resources in the WWC. However, given the practice of natural resource degradation activities that occur in the area, degrading the water sources, capacity building is needed to enable beneficiaries to organize themselves to maintain and preserve these water points collectively. In the same vein, building the capacity of stakeholders on landscape governance approaches adapted to their context, such as ILAs, could help mobilize stakeholders better and improve collaboration and synergy of actions between them.

Third, the CREMA management bodies need to be revitalized. All three CREMAs in the study area lack the financial and technical resources needed for their functioning. Technical and financial support from the state for at least three years would considerably improve the functioning of the CREMA bodies and allow the system to be established more firmly in the communities. This requires logistical support (means of transport, protection equipment), financial resources for the organization of meetings and patrols, and training of CRMC and CEC members in natural resource governance and project design. The Forestry Commission and its partners could play a key role in such initiatives.

Fourth, a functional monitoring and evaluation mechanism is essential to improve the functioning of CREMAs. Stakeholder involvement is key in ensuring a greater sense of ownership of the CREMA concept at the community level.

### Methodological Reflections

Social research plays a crucial role in conservation as any conservation initiative has a social character (Teel et al. 2018; Zabala et al. 2018). Researchers and practitioners have widely recognized the need to consider the human factor in natural resource conservation processes (Carmenta et al. 2017; Zabala et al. 2018). For these reasons, Q-methodology is recognized for its relevance to perception and discourse analyses in natural resource governance and conservation contexts. However, it has been criticized for its subjectivity and reliance on researcher interpretations (Sumberg et al. 2017; Zabala et al. 2018).

Another weakness concerns the participant selection criteria. The requirement to be able to read and write can lead to the exclusion of relevant actors from the research process, as was the case in this study. The elders and pastoralists were not included in the ranking of the Q-sets, although their views were considered as they participated in the focus group discussions. This exclusion is a limitation in that the results do not allow knowing which discourses they are linked to, and this does not help in including them in implementing a conservation initiative or an ILA. Similarly, this may result in a gender bias as illiteracy is higher among women than men (Fairweather and Swaffield 2002; Jones and Chant 2009; Naspetti et al. 2016; Takyi et al. 2021; Sáenz de Tejada et al. 2021). However, there is a growing literature on using pictures to overcome that challenge (e.g.,Webler et al. 2009; Milcu et al. 2014; Naspetti et al. 2016; Sáenz de Tejada et al. 2021), but time and resource limitations did not allow me to develop it.

Also, due to time constraints and restrictions related to the COVID-19 pandemic, this study could only consider three CREMAs out of the six that make up the WWC. Furthermore, the WWC landscape goes beyond the CREMAs. Several communities were therefore excluded from the study, even though their contributions (perceptions, perspectives) could have influenced the results obtained, particularly regarding common entry points. Therefore, it would be interesting and useful to conduct a study on the CREMAs that I was unable to explore, as well as on other communities outside the CREMAs, to understand the perspectives of a broader population representation. In addition, a more comprehensive study, which considers all communities in the WWC at once, could provide comparative results to our own.

This study has shown the practicality of Q-methodology. Indeed, the merit of Q lies in its simplicity in collecting data and the possibility of analyzing subjective (qualitative) data through a quantitative approach. Q-methodology is recognized for its flexibility in collecting and analyzing data (Lundberg et al. 2020). Specifically, the phase of classifying the statements is similar to a manual work exercise (cutting out the cards and sticking them on the Q-grid), which created fascination and excitement among the respondents. This facilitated interaction between the research team and the respondents and reduced the stress of participating in the study.

## Conclusions

The WWC landscape is a source of opportunities and a space for biodiversity conservation for a range of stakeholders. The study shows that these different actor groups, namely local landscape users, practitioners engaged in conservation and sustainable resource use, and private actors, have diverging interests, and there are conflicting claims, including between conservation and development. This plurality of stakeholders implies a diversity of perceptions on the major issues affecting the landscape and its governance and the solutions that could ensure the sustainability of landscape resources. The main discourses that emerged from the study, related to challenges of the WWC landscape and its governance, focus on destructive livelihood activities, constrained livelihoods, and a weak governance system. Concerning the possible solutions, the discourses suggest balancing livelihoods with conservation, strengthening landscape governance through the CREMA initiative, and raising knowledge awareness but forget about stakeholder mobilization. However, shared perceptions of problems and solutions form the basis of identifying common concern entry points for implementing ILAs. Regarding the problems, the consensus views are related to the siltation and drying up of water bodies and the financial difficulties that affect the CREMA initiative. As for solutions to improve landscape governance, the shared perceptions are associated with the need for livelihood support and the necessity to implement a monitoring and evaluation mechanism in the CREMA governance system.

Thus, this study provides insights into how the local stakeholders of the WWC perceive the problems of the landscape and the potential solutions and the consensual views among them. This provides a basis for identifying common concern entry points of use to conservation initiatives and efforts to implement ILAs. From the findings of this study, I conclude that perceptions are valuable data sources to guide landscape governance and promote informed decision-making.

### Supplementary Information

Supplementary Material

## Footnotes

1 This study uses stakeholders and actors interchangeably. In the ILA literature, stakeholders are understood as “individuals, groups and organizations who are affected by or can affect (…) policy, development and natural resource management” (Reed et al. 2009: 1933). Actors are often understood as more active stakeholders who are “able to influence a decision” (Marques et al. 2020: 3).

2 Ghanaians do not often use the word “problems”, but use “challenges” instead.

3 https://www.cifor-icraf.org/colands/.

4 A community is qualified as “large” not only based on its surface area but also based on the presence of an important market and social infrastructure (school, hospital), which small villages do not have.

5 Interview held in Bolgatanga on 27/10/2021.

6 Interview held in Bolgatanga on 04/10/2021.

7 Interview held in Nakong on 19/05/2021.

8 Interview held in Paga on 12/10/2021.

9 Commonly, research participants used the term “education” for capacity building or “educating communities”.

10 Focus group held in Kunyinsa (Kanjarga) on 22/05/2021.

11 “*Bhungroo* is a water management system that injects and stores excess rainfall underground and lifts it out for use in dry spells. Adoption of this technology has decreased salt deposits on soil and increased fresh water supply, saving farmers from drought.” https://cop23.unfccc.int/climate-action/momentum-for-change/women-for-results/bhungroo.

12 Interview held in Nakong on 19/05/2021.

13 Interviews held in Kunyinsa on 21/05/2021.

14 Interview held in Bolgatanga on 27/10/2021.

15 Interview held in Nakong on 19/05/2021.

16 https://www.cifor.org/publications/pdf_files/Books/2020-COLANDS-05.pdf.

17 Ingredient from *Parkia biglobosa* seeds.
