# Supplementary material for: Stakeholder Perceptions on Landscape Governance in Northern Ghana: A Q-Study to Identify Common Concern Entry Points for Integrated Landscape Approaches
Source: Environ Manage. 2023 Sep 30;74(1):31–51. doi: 10.1007/s00267-023-01881-2 (PMC11208230; doi:10.1007/s00267-023-01881-2)
Supplement: Supplementary file 1 — Supplementary Material [file 267_2023_1881_MOESM1_ESM.docx]

**Supplementary material**

**Appendix 1: Semi-structured interview guides**

**CHIEFS INTERVIEW**

**Note:** *This research is a collaborative project of the University of Amsterdam and the Centre for International Forestry Research in Northern Ghana (Western Wildlife Corridor). The questionnaire is to collect research data that will be used for academic purposes and project implementation. Respondents have the right to remain anonymous and withdraw from the interview at any time.*

**Identification of the interviewee**

Name of territory/ village:

CREMA Name:

Name of interviewee:

Contacts:

**Traditional management of natural resources (RQ3)**

1. How is natural resources management organized in your territory according to traditional rules?

2. What taboos are in place with regard to the use of natural resources?

**Decision-making process at the chief territory level (RQ3)**

3. Who is involved, and how are decisions made with regard to:

| Farmland allocation? | The use of trees? | Hunting? | The use of grazing land? | Mining? | The use of water? | The use of? |
| --- | --- | --- | --- | --- | --- | --- |
|  |  |  |  |  |  |  |

4. How do you ensure equitable participation in decision-making, considering gender and different landscape users?

**Balancing the interest of conservation, livelihoods, and development (RQ3)**

5. How do you balance conservation, livelihood, and development interests in your territory?

6. What are the major challenges you face in terms of natural resource governance?

**Decision-making process and landscape governance at CREMA level (RQ3)**

7. Are you involved in decision-making on landscape governance at the CREMA level? What role do you play?

8. How do you see the current way to govern the landscape/CREMA?

**Do you have any final comments or suggestions to share?**

***Thank you for your participation in this research!!***

**KEY RESPONDENT INTERVIEWS (reps of CEC/CRMC)**

**Note:** *This research is a collaborative project of the University of Amsterdam and the Centre for International Forestry Research in Northern Ghana (Western Wildlife Corridor). The questionnaire collects research data that will be used for academic purposes and project implementation. Respondents have the right to remain anonymous and withdraw from the interview at any time.*

**Identification**

CEC or CRMC:

CREMA Name:

Name of interviewee :

**I- Perceptions of landscape actors on landscape issues, dynamics, and solutions**

**I-1- Perceptions of landscape in general**

I-1-1- What does ‘landscape’ mean to you?

I-1-2- What is the extent of the landscape that is relevant to you?

**I-2- Perceptions of landscape issues**

I-2-1- What do you see as the three main challenges/problems in the landscape in order of importance?

I-2-2-Who/what causes these problems? (ask per problem mentioned under I.-2-1)

**I-3- Perceptions of landscape changes**

I-3-1- Have you noticed a change in the state of the landscape in the last 20, 10, or 5 years? YES NO

I-3-2-How these changes manifested in terms of:

| Vegetation cover? | Example: |
| --- | --- |
| Wildlife? | Example: |
| Products collected from the wild (NTFPs)? | Example: |
| Water availability? | Example: |

I-3-3- What do you think is the cause of these changes?

I-3-4- What are the consequences of these changes on your livelihood?

**I-4- Perception of solutions and landscape governance**

I-4-1- What do you think needs to be done to solve the problems/challenges mentioned?

I-4-2- What do you think about landscape governance?

I-4-3- What is, in your view, the best way to govern the landscape?

I-4-4- Who should play a role in landscape governance and how?

**II- INCLUSIVENESS OF DECISION-MAKING (RQ3)**

**II-1- Taking gender into account (RQ3)**

II-1-1- How many CEC/CRMC meetings do you organize per year?

II-1-2- Are **women** involved in the meeting? YES NO

- If not, why is that the case?
- Are they consulted prior to the meeting? How?
- If yes, how do you rate the influence of women in the decision-making process on a scale from 1-5?

1 = very little 2 = little 3 = neither little or much 4= much 5 = very much

- Can you explain your rating?

II-1-3- Is the **youth** involved in the meeting? YES NO

- If not, why is that the case?
- Are they consulted prior to the meeting? How?
- If yes, how do you rate the influence of youth in the decision-making process on a scale from 1-5?

1 = very little 2 = little 3 = neither little or much 4= much 5 = very much

- Can you explain your rating?

II-1-4- Are the **elderly** involved in the meeting? YES NO

- If not, why is that the case?
- Are they consulted prior to the meeting? How?
- If yes, how do you rate the influence of the elderly in the decision-making process on a scale from 1-5?

1 = very little 2 = little 3 = neither little or much 4= much 5 = very much

- Can you explain your rating?

**II-2- Consideration of socio-professional groups (RQ3)**

II- 2-1-Which of the following **natural resource user groups** are involved in the CEC/CRMC meetings?

- Farmers
- Fishermen
- NTFP collectors
- Herders
- All of them

If any of those is not included, why is that the case?

II- 2-1- Which institutions take part in your decision-making process? Why?

**III- INTERACTIONS AND SYNERGIES**

**Collaboration among stakeholders (fill in the table below)**

- With which organizations/actors does your committee collaborate to manage the landscape?
- What is the purpose of your collaboration?
- How strong is the collaboration?

Table 2: Stakeholders interactions and synergies scoring

Name of organization/actor interviewed……………………………………………………………...

| **Name of organization** | **Purpose of collaboration with this organization?**  Capacity building, sensitization, information exchange, financial support, training, project implementation partnership, etc. | **How strong is your collaboration?**  1= very weak  2= weak  3= moderate  4= strong  5= very strong | **How satisfactory is the collaboration?**  1= very unsatisfactory  2= unsatisfactory  3= neither unsatisfactory nor satisfactory  4= satisfactory  5=very satisfactory |
| --- | --- | --- | --- |
| Formal actors (public organisations, NGOs, research centres, …) | | | |
|  |  |  |  |
| Informal actors (local association of farmers, breeders, forest operators | | | |
|  |  |  |  |
| What are the main reasons for your institution to collaborate effectively?  What are the main reasons for your institution to not effectively collaborate?  What are solutions for your institution to improve the collaboration with the other structures, according to you | | | |

**Do you have any final comments or suggestions to share?**

**Thank you for your participation in this research!!**

**KEY RESPONDENT INTERVIEWS**

**(reps of government and non-governmental organizations)**

**Note:** *This research is a collaborative project of the University of Amsterdam and the Centre for International Forestry Research in Northern Ghana (Western Wildlife Corridor). The questionnaire collects research data that will be used for academic purposes and project implementation. Respondents have the right to remain anonymous and withdraw from the interview at any time.*

**Identification**

Name of organization:…………………………………………………….. …………….…………………

District Name:……………………………………………Region of………………………………………

Name of interviewee (facultative):………………………………………………………………………...

**I- Overview of landscape actors and interest (RQ1)**

**I-1- Governance actors**

I-1-1-What is the mission of your organization?

Conserving nature

Improving livelihoods

Forest exploitation

Other……………………………………………………………………………...................................

I-1-2-What kind of activities does your organization undertake in the CREMA?

Capacity-building in conservation

Development of income-generating activities

Logging (NTFP, charcoal,...)

Other…………………………………………………………………………………………………..

I-1-3-Which other organizations do you consider important in the CREMA management/ governance?

| Government organizations |  |
| --- | --- |
| NGOs |  |
| Local actors |  |

**II- PERCEPTIONS OF LANDSCAPE ACTORS ON LANDSCAPE ISSUES, DYNAMICS, AND SOLUTIONS (RQ1)**

**II-1- Perceptions of landscape in general (RQ1)**

II-1-1- What does ‘landscape’ mean to you?

II-1-2- What is the extent of the landscape that concerns you?

**II-2- Perceptions of landscape issues (RQ1)**

II-2-1- What do you see as the three main challenges/problems in the WWC **landscape** in order of importance?

II-2-2-Who/what causes these problems? (ask per problem mentioned under I.-2-1)

II-2-3- What do you consider the three main challenges related to **land uses**?

| Challenge in order of importance | Causes/Actors | What are the consequences? |
| --- | --- | --- |
| 1. |  |  |

II-2-4- What do you consider the three main challenges related to **nature conservation?**

| Challenge in order of importance | Causes/Actors | What are the consequences? |
| --- | --- | --- |
| 1. |  |  |

**II-3- Perceptions of landscape changes (RQ1)**

**II-4- Actions undertaken and conservation benefits (RQ1)**

II-4-1-What actions are you undertaking with regard to nature conservation or the conservation of natural resources?

II-4-2-What are the benefits of biodiversity conservation for local communities?

**II-5- Perception of solutions (RQ1)**

II-5-1- What do you think needs to be done to solve problems/challenges related to:

- ***Conflicting land uses?***
- ***Nature conservation?***

II-5-2- What are the limitations of putting measures in place related to:

- ***Conflicting land uses?***
- ***Nature conservation?***

II-5-3- What is, in your view, the best way to govern the landscape in general and CREMAs in particular?

II-5-4- Who should play a role and How?

**III- DECISION MAKING-PROCESS (RQ3)**

**III-1- Actors and process of decision-making (RQ3)**

III-1-How does your organization participate in CREMA decisions making processes?

III-2-Who else participates in these decisions making processes as a stakeholder?

III-3-How are women/youths/elderly taken into account in the decision-making process?

III-4-Do you think that there are one or more actors who should be involved in decision-making but are not? NO YES (Who?)

III-5-If any, do you know why they are currently not included?

III-6-Is there, in your opinion, any stakeholder whose voice counts more than others in decision-making or to whom is listened more than to others during the meetings? If any,

- Which stakeholder?
- Why does this stakeholder have more weight in decision-making?
- How does their influence affect the decision-making process?

**IV- EXISTING PLATFORMS**

**IV-1-Type of existing platforms** (*Fill in the table below by responding to questions*)

IV-1-1- Which platforms exist where the CREMA actors meet and make decisions?

Ask for each platform mentioned:

IV-1-2-Can you categorize this platform (project based (temporary) or institutionalized (permanent)?

IV-1-3- How many times do you meet a year, depending on the platform?...............................

IV-1-4- Are there particular periods for meetings? (or do you meet when there is a need?)

IV-1-5- Which organization leads the meetings in the platform?

IV-1-6 What topics are discussed at the platform meetings?

IV-2-7- What is the potential of this platform for the governance of natural resources?

IV-2-8- What challenges does this platform face in the governance of natural resources?

IV-2-9- Which platform seems to bring together all the main stakeholders

Table1: Existing platforms

| Platform name | Status (temporary or permanent) | Annual meeting frequency | Meeting period | | Platform leader | Topics discussed | Potential for resource governance | Challenges related to its functioning |
| --- | --- | --- | --- | --- | --- | --- | --- | --- |
|  |  |  | |  |  |  |  |  |

**V-INTERACTIONS AND SYNERGIES**

**Collaboration among stakeholders (fill in the table below)**

- With which organizations/actors does your organization collaborate to manage the landscape?
- What is the purpose of your collaboration?
- How strong is the collaboration?

Table 2: Stakeholders interactions and synergies scoring

| **Name of organization** | **Purpose of collaboration with this organization?**  Capacity building, awareness raising, information exchange, financial support, training, project implementation partnership, etc. | **How strong is your collaboration?**  1= very weak  2= weak  3= moderate  4= strong  5= very strong | **How satisfactory is the collaboration?**  1= very unsatisfactory  2= unsatisfactory  3= neither unsatisfactory nor satisfactory  4= satisfactory  5=very satisfactory |
| --- | --- | --- | --- |
| Formal actors (Public organisations, NGOs, research centres, …) | | | |
|  |  |  |  |
| Informal actors (local association of farmers, breeders, forest operators | | | |
|  |  |  |  |
| What are the main reasons for your institution to collaborate effectively?  What are the main reasons for your institution to not effectively collaborate?  What are solutions for your institution to improve the collaboration with the other structures, according to you? | | | |

**Do you have any final comments or suggestions to share?**

**Thank you for your participation in this research!!**

**Appendix 2: Focus Group Discussion Protocols**

**FOCUS GROUP DISCUSSION (resource user groups/farmers, herders, forest operators)**

1. **Questions:**

- **What do you think are the problems affecting your landscape?**
- **How do you think these problems can be solved?**
- **What do you think about the governance system of your landscape/CREMA?**

1. **Follow-up Questions:**

**I- Overview of landscape actors and interest**

**I-1- Resource users**

I-1-1- How do you make use of the landscape resources?

Agriculture NTFP Collection Charcoal production Herding/Grazing Other

I-1-2- Which other user groups do you distinguish? *(in order of importance)*

**II- Perceptions of landscape actors on landscape issues, dynamics, and solutions**

**II-1- Perceptions of landscape issues (RQ1)**

II-1-1- What do you see as the Five main challenges/problems in your landscape in order of importance?

II-1-2-Who/what causes these problems? (ask per problem mentioned under I.-1-1)

**II-2- Perception of solutions**

II-2-1- What do you think needs to be done to solve these problems/challenges?

**II-3- Perceptions of landscape changes (RQ1)**

II-3-1- What kind of changes have you noticed in the state of the landscape in the last 20, 10, or 5 years?

| Vegetation cover? | Example: |
| --- | --- |
| Wildlife? | Example: |
| Products collected from the wild (NTFPs)? | Example: |
| Water availability? | Example: |
| Other | Example: |

II-3-2- What do you think is the cause of these changes?

II-3-3- What are the consequences of these changes on your livelihoods?

**II-4- Perception of conservation beneficiaries (RQ1/2)**

II-4-1- What are the benefits of nature /natural resources conservation you know?

II-4-2- In your opinion, how do you benefit from the conservation of natural resources and biodiversity?

**II-5- Perception of landscape governance**

II-5-1-What role do you play in the CREMA governance?

II-5-2-How do you see the current way to govern the landscape/CREMA

II-5-3- What is, in your view, the best way to govern the landscape/CREMA?

II-5-4- Who should play a role and How?

**III- DECISION MAKING-PROCESS (RQ3)**

**III-1- Involvement of local actors in decision-making (RQ3)**

III-1-How do you participate in decision-making about conservation or development in your CREMA?

III-2- In which decision-making body are you involved?

III-3-How satisfied are you with the decisions made?

(1) Very unsatisfactory (2) Unsatisfactory (3) Unsatisfactory nor satisfactory (4) Satisfactory (5) Very satisfactory

Can you justify your answer?

III-4-Who do you know that takes part in the decision-making, and who can influence decisions?

III-5-How do you think the decision-making process should be done?

1. **EXIT QUESTION**

**Do you have any final comments or suggestions to share?**

**Thank you for your participation in this research!**

**FOCUS GROUP DISCUSSION: Youths, Women, Elder**

1. **Questions:**

- **What do you think are the problems affecting your landscape?**
- **How do you think these problems can be solved??**
- **What do you think about the decision-making process related to the governance of your landscape/CREMA?**

1. **Follow-up Questions:**

**I- Perceptions of landscape actors on landscape issues, dynamics, and solutions**

**I-1- Perceptions of landscape issues (RQ1)**

I-1-1- What do you see as the Five main challenges/problems in your landscape in order of importance?

I-1-2-Who/what causes these problems?

**I-2- Perception of solutions (RQ1)**

I-2-1- What do you think needs to be done to solve these problems/challenges?

**II- PERCEPTION OF LANDSCAPE CHANGES (RQ1/2)**

II-1- What kind of changes have you noticed in the state of the landscape in the last 20 years?

| Vegetation cover? | Example: |
| --- | --- |
| Wildlife? | Example: |
| Products collected from the wild (NTFPs)? | Example: |
| Water availability? | Example: |
| Other | Example: |

II-2- What do you think is the cause of these changes?

II-3- What are the consequences of these changes on your livelihoods?

**III- PERCEPTION OF CONSERVATION BENEFICIARIES (RQ1/2)**

II-5-1-What are the benefits of nature /natural resources conservation you know?

II-5-2-In your opinion, how do you benefit from the conservation of natural resources and biodiversity?

**IV- PERCEPTION OF LANDSCAPE GOVERNANCE**

IV-1-What role do you play in the CREMA governance?

IV-2-How do you see the current way to govern the landscape/CREMA

IV-3- What is, in your view, the best way to govern the landscape/CREMA?

IV-4- Who should play a role and How?

**V- DECISION MAKING-PROCESS (RQ3)**

V-1- Involvement of local actors in decision-making (RQ3)

V-1-How do you participate in decision-making about conservation or development in your CREMA?

V-2- In which decision-making body are you involved?

V-3- How satisfied are you with the decisions made?

- Very unsatisfactory (1)
- Unsatisfactory (2)
- Unsatisfactory nor satisfactory (3)
- Satisfactory (4)
- Very satisfactory (5)

Can you justify your answer?

V-4-Who do you know that takes part in the decision-making, and who can influence decisions?

V-1- How do you think the decision-making process should be done?

1. **EXIT QUESTION**

**Do you have any final comments or suggestions to share?**

**Thank you for your participation in this research!!**

**Appendix 3: Distinguishing and consensus statements/ research question 1 (RQ1)**

| Num. of statement | Dist. and consensus | f1_f2 | sig_f1_f2 | f1_f3 | sig_f1_f3 | f2_f3 | sig_f2_f3 |
| --- | --- | --- | --- | --- | --- | --- | --- |
| 1 | Distinguishes f3 only | -0.2 |  | 2.1 | 6* | 2.4 | 6* |
| 2 | - | 0.4 |  | -0.2 |  | -0.6 | * |
| 3 | Consensus | 0.1 |  | 0.4 |  | 0.3 |  |
| 4 | - | -0.2 |  | 0.5 |  | 0.7 | * |
| 5 | Distinguishes all | -2.8 | 6* | -1.3 | *** | 1.5 | 6* |
| 6 | Distinguishes f1 only | -1.6 | 6* | -1.3 | *** | 0.3 |  |
| 7 | Distinguishes all | -0.7 | ** | -2.8 | 6* | -2.1 | 6* |
| 8 | - | 0.5 |  | 0.6 | * | 0.1 |  |
| 9 | Distinguishes f3 only | -0.3 |  | 0.9 | ** | 1.2 | *** |
| 10 | Distinguishes f3 only | 0.3 |  | -1.6 | 6* | -1.8 | 6* |
| 11 | Distinguishes f1 only | 1.4 | 6* | 1.7 | 6* | 0.3 |  |
| 12 | - | 0.3 |  | -0.5 |  | -0.8 | * |
| 13 | - | 0.6 | * | 0.2 |  | -0.4 |  |
| 14 | - | 0.1 |  | 0.7 | * | 0.5 |  |
| 15 | Distinguishes all | 2.6 | 6* | 0.8 | ** | -1.8 | 6* |
| 16 | Consensus | -0.4 |  | -0.2 |  | 0.1 |  |

Source: Field data, 2021.

**Appendix 4: Distinguishing and consensus statements/ research question 2 (RQ2)**

| Num. of statement | Dist. and consensus | f1_f2 | sig_f1_f2 | f1_f3 | sig_f1_f3 | f2_f3 | sig_f2_f3 |
| --- | --- | --- | --- | --- | --- | --- | --- |
| 1 | Distinguishes f3 only | -0.38 |  | -2.1 | 6* | -1.71 | 6* |
| 2 | Distinguishes all | 2.56 | 6* | 1.6 | 6* | -0.96 | *** |
| 3 | Consensus | -0.40 |  | -0.5 |  | -0.09 |  |
| 4 | Distinguishes f3 only | 0.05 |  | 1.2 | *** | 1.17 | *** |
| 5 | - | 0.38 |  | -0.3 |  | -0.63 | * |
| 6 | - | -0.07 |  | -0.6 | * | -0.49 |  |
| 7 | Distinguishes f3 only | 0.24 |  | 1.3 | 6* | 1.04 | *** |
| 8 | Distinguishes all | 0.69 | * | 1.9 | 6* | 1.21 | *** |
| 9 | Distinguishes f3 only | 0.43 |  | -1.5 | 6* | -1.93 | 6* |
| 10 | Distinguishes f1 only | -1.57 | 6* | -1.2 | *** | 0.32 |  |
| 11 | Consensus | -0.49 |  | -0.5 |  | -0.03 |  |
| 12 | Distinguishes f3 only | -0.10 |  | 0.8 | ** | 0.87 | ** |
| 13 | Distinguishes f2 only | -0.95 | *** | 0.3 |  | 1.27 | *** |
| 14 | Distinguishes f3 only | 0.40 |  | -1.2 | *** | -1.58 | 6* |
| 15 | Distinguishes all | 0.76 | ** | 1.4 | 6* | 0.62 | * |
| 16 | Distinguishes all | -1.58 | 6* | -0.7 | * | 0.91 | *** |

Source: Field data, 2021.
